# Effectiveness of a multifaceted intervention on reducing non-guideline-concordant prescribing of calcium and vitamin D analogues after total thyroidectomy

**DOI:** 10.3389/fphar.2026.1795773

**Published:** 2026-03-19

**Authors:** Yue Xie, Furong Han, Shizhi He, Jiawei Wang

**Affiliations:** 1 Department of Pharmacy, Beijing Tongren Hospital, Capital Medical University, Beijing, China; 2 Department of Head and Neck Surgery, Beijing Tongren Hospital, Capital Medical University, Beijing, China

**Keywords:** clinical pharmacist, hypocalcemia, medication therapy management, quality improvement, thyroidectomy

## Abstract

**Background:**

Postoperative hypocalcemia is a common complication of total thyroidectomy. Due to a lack of standardized perioperative management protocols, there is often a wide variation in clinical practice, resulting in non-guideline conforming calcium and vitamin D analogue (NGC-CaVD) prescribing. This can lead to issues with patient safety and recovery. Objective To determine if a multi-faceted intervention could reduce NGC-CaVD after total thyroidectomy, assess safety outcomes, and identify factors associated with inappropriate prescribing.

**Methods:**

This retrospective observational cohort study included 629 adults undergoing total thyroidectomy (322 pre-intervention, 307 post-intervention). The intervention bundle included: (1) developing an institutional guideline, (2) conducting staff education, (3) pharmacist-led medication reviews with structured recommendations, (4) medication reconciliation, and (5) collaborative rounds. The primary outcome was the rate of NGC-CaVD. Safety outcomes included the incidence of symptomatic hypocalcemia and length of hospital stay.

**Results:**

The multifaceted intervention significantly reduced the overall NGC-CaVD rate from 45.96% in the pre-intervention group to 23.45% in the post-intervention group (*p* < 0.01). Results Improvements were also seen in specific areas: inappropriate calcium preparation selection (8.07% *versus* 2.93%, *p* < 0.01), calcium dosage (23.91% *versus* 11.73%, *p* < 0.01), vitamin D analogue dosage (30.75% *versus* 6.84%, *p* < 0.01), and delayed IV to oral switch (4.97%–1.95%, *p* = 0.04). The per-group NGC-CaVD cost decreased from RMB 17.34 (IQR: 0, 19.56) to RMB 4.42 (IQR: 0, 0) (p < 0.01). The main barrier influencing NGC-CaVD was physician-related factors, including excessive concern for the risk of postoperative hypocalcemia. The rate of symptomatic hypocalcemia remained unchanged (6.83%–5.86%, *p* = 0.62) as did mean hospital stay (7.16 ± 2.70 days vs. 7.21 ± 2.65 days, *p* = 0.75). Multivariable analysis identified that longer surgical times (adjusted OR: 1.012, 95% CI: 1.007–1.035, *p* = 0.023) and longer lengths of stay (adjusted OR: 1.128, 95% CI: 1.008–1.262, *p* = 0.036) were independent predictors of NGC-CaVD.

**Conclusion:**

The implementation of a multi-faceted intervention improved guideline adherence for postoperative prescribing of calcium and vitamin D analogues without affecting symptomatic hypocalcemia and length of stay. Additionally, the model provides a framework for the standardization of post-thyroidectomy hypocalcemia management and the improved appropriateness of medication prescribing.

## Introduction

Thyroidectomy is one of the most commonly performed surgical procedures for various thyroid pathologies, including thyroid cancer, hyperthyroidism, and large goiters. Despite significant advances and improved surgical techniques, postoperative hypocalcemia remains one of the more common complications reported, with incidence rates ranging from 12% to 64% (Eismontas et al., 2018). Although most cases of hypocalcemia are transient in nature, approximately 2.7%–9.5% of patients will develop permanent hypocalcemia (Lin et al., 2017). Postoperative hypocalcemia can affect patients clinically, leading to clinical manifestations such as perioral numbness, paresthesias, and carpopedal spasms, and in severe cases, may lead to life-threatening hyperacute symptoms such as laryngospasm or seizures. These adverse effects not only pose risks to patient safety and quality of recovery, but also significantly increase the risk of prolonged stay and healthcare costs.

The major contributor to post-thyroidectomy hypocalcemia occurs due to damage to the parathyroid glands during the procedure; causes may include accidental resection, vascular compromise, thermal injury, or functional suppression. Many patient and procedures based factors are also known to increase the risk of hypocalcemia, such as an increased body mass index (BMI >40 kg/m2), Graves’ disease, completing a total thyroidectomy or central lymph node dissection, and low vitamin D levels (Pasieka et al., 2022). Given that both assessment and management of risk factors take place in various clinical settings, a team-based, multi-disciplinary approach is necessary from the outset to improve patient outcomes, as each approach to pre-operative planning, and working together post-operatively collectively improves definitions and potential complications of hypocalcemia.

To address post-operative hypoparathyroidism, clinical practice guidelines have been developed from professional societies such as the American Thyroid Association and the British Association of Endocrine and Thyroid Surgeons (Orloff et al., 2018; British Association of Endocrine and Thyroid Surgeons BAETS, 2012). These guidelines provide some foundational recommendations for assessment and management, including pre-operative assessment of low serum calcium and 25-hydroxy vitamin D levels followed by preventative correction, intra-operative approaches to protect parathyroid glands, the need for early determination of serum calcium and parathyroid hormone (PTH) post-operatively to plan a guided stratified calcium and/or vitamin D analogue replacement. However, the guidelines tend to stop short and provide a comprehensive overview of hypocalcemia/hypoparathyroidism management for post-operative recovery and are less specific about the total pre-operative management pathway that could be put in place to optimize outcomes for patients with hypocalcemia. The consequence of the lack of highly specific, universally adopted standardized management protocol has been there is high variability in practice and care variations both across institutions and individual surgeon practices. This variability is seen primarily in three areas of practice: 1) how often biochemical parameters are monitored; 2) whether prophylactic calcium and vitamin D supplementation is used and the timing; and 3) how to implement stratified treatment for patients with hypocalcemia.

In our clinical practice, we found that more than 50% of post-thyroidectomy patients who were treated with inappropriate calcium and vitamin D analogues during hospitalization, including inappropriate medication selection, dosing, and surveillance, which may significantly impair calcium homeostasis. These findings highlight some fundamental deficits in management approaches and the need for systems-based clinical frameworks.

To address the above issues, we designed and implemented a collaborative co-management model between surgeons and pharmacists to provide perioperative management strategies for thyroidectomy. This study evaluates the effectiveness of the management by comparing the rationality of hypocalcemia drug therapy before and after the implementation of these measures. The safety of the management is assessed by monitoring the incidence of symptomatic hypocalcemia. Additionally, we identified the risk factors associated with improper drug use in order to clarify the barriers to achieving optimal care. This study aims to establish evidence-based, patient-centered perioperative management for thyroidectomy, with the goals of minimizing inappropriate drug therapy and improving clinical outcomes after thyroid surgery.

## Methods

### Study design and setting

This single-center, observational retrospective cohort study was conducted at the Head and Neck Surgery Ward of Beijing Tongren Hospital, Capital Medical University. This hospital is considered one of the major tertiary academic health centers with 1,759 inpatient beds.

#### Study population

All adult patients (≥18 years of age) who underwent total thyroidectomy at Beijing Tongren hospital were included in the present study between the dates of 1 November 2024 to 31 October 2025. Patients were excluded according to the following criteria: (a) Preoperative abnormal levels of PTH, calcium, phosphorus, magnesium, or thyroid-stimulating hormone (TSH); (b) hypoproteinemia; (c) history of neck irradiation or prior central neck compartment surgery; (d) Graves’ disease; (e) parathyroid disorders; (f) granulomatous diseases impacting calcium metabolism; (g) severe renal insufficiency; (h) severe hepatic insufficiency; (i) heart failure; (j) Other endocrine diseases known to significantly disrupt calcium homeostasis.

The research population was divided into two groups: a pre-intervention group and a post-intervention group. The pre-intervention cohort included patients who underwent total thyroidectomy from 1 November 2024 to 30 April 2025, during which no standardized institutional protocol existed for perioperative hypocalcemia management. The multifaceted intervention was developed during a 2-month preparatory phase (March 1 to 30 April 2025), followed by full implementation from May 1 to 31 October 2025. The preparatory phase involved finalizing the institutional protocol and conducting initial educational sessions. The post-intervention cohort comprised patients treated from 1 May 2025 to 31 October 2025.

### Multifaceted intervention

The intervention bundle, developed based on existing evidence and guidelines, consisted of five core components designed to standardize and improve the quality of care across the perioperative pathway.

#### (a) Development of an institutional clinical practice guideline

**FIGURE 1:**
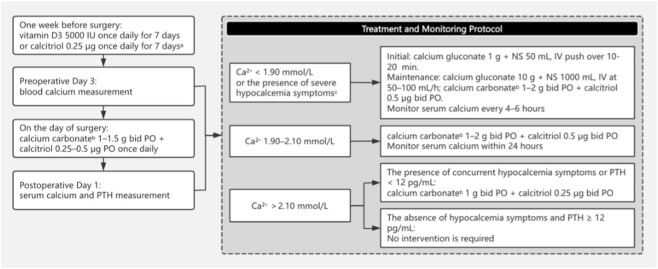
Guidelines for the management of hypocalcemia following total thyroidectomy. a: Contraindications to vitamin D_3_ supplementation include hypercalcemia, hyperphosphatemia, hypervitaminosis D, severe renal or hepatic insufficiency, hyperthyroidism, granulomatous disorders, nephrolithiasis, known hypersensitivity to vitamin D, and concurrent daily intake exceeding 800 IU of vitamin D or 1,200 mg of elemental calcium from all supplemental sources. b: In patients with impaired absorption of oral calcium carbonate, such as those with a history of gastric bypass surgery or those receiving proton pump inhibitors (PPIs) or H_2_-receptor antagonists, alternative calcium salts such as calcium citrate may be preferred due to their better bioavailability under low-acid conditions. c: Severe hypocalcemia symptoms include laryngospasm, carpopedal spasm, seizures, and QT interval prolongation.

A multi-disciplinary group of head and neck surgeons, endocrinologists, pharmacists, and nurses developed an institutional clinical practice guideline regarding post-thyroidectomy management of perioperative hypocalcemia, as shown in Figure 1. The perioperative hypocalcemia management protocol is as follows: All patients receive vitamin D3 or calcitriol supplementation starting 7 days preoperatively, except those with contraindications to vitamin D3. Routine preoperative assessment of serum 25-hydroxyvitamin D levels is not performed. Serum calcium is measured 3 days before surgery; on the first postoperative day, both serum calcium and PTH levels are assayed. The dosing regimen of calcium and calcitriol is individually titrated based on serum calcium concentrations, PTH levels, and clinical manifestations, with the frequency of serum calcium monitoring dynamically adjusted accordingly.

The guideline was developed, through the use of the best available evidence, and reviewed, as a formal draft, by the Medical Quality Department before being approved for implementation. The guideline is comprehensive in its description of managing perioperative hypocalcemia and includes information pertaining to the pre-and post-operative vitamin D replacement criteria, monitoring and assessing methods for serum calcium levels and parathyroid hormone levels, patient risk classification for hypocalcemia occurrence, and dosing guidance for calcium and active vitamin D analogue supplementation. The final guideline has been posted on the hospital intranet for all to read and has been mandated for use by all head and neck surgeons in the institution.

#### (b) Educational sessions for the clinical team

Multidisciplinary educational sessions were held for the head and neck surgeons, surgical residents, nurses and clinical pharmacists to ensure complete understanding and adherence to the new hypocalcemia guideline. Educational sessions were conducted as follows: i) two 2-h interactive workshops for all head and neck surgeons and surgical residents, covering guideline rationale, risk stratification, and case-based discussions; ii) three 1-h training sessions for nurses and clinical pharmacists, focusing on the content of therapy monitoring, medication review protocols, and documentation of recommendations; iii) distribution of pocket cards and posters displaying the management algorithm to all participating clinical staff; iv) monthly 30-min refresher sessions held during the study period. Attendance was mandatory and documented.

#### (c) Pharmacist medication review/recommendations

Pharmacists played a principal role in the care continuum by proactively reviewing the medical records of surgical patients. They utilized the institutional guideline to provide guidance to Head and Neck Surgeons regarding the most appropriate perioperative hypocalcemia management strategies. Medication reviews by pharmacists were conducted for all patients undergoing thyroidectomy in the perioperative period in the Head and Neck Surgery ward. The review process included: i) Verification of risk stratification based on laboratory values and clinical symptoms; ii) Assessment of calcium and vitamin D analogue prescribing against guideline criteria; iii) Documentation of recommendations in the electronic medical record using a standardized template; iv) Direct communication with the primary surgical team for non-concordant prescriptions. Recommendations were categorized as: Dose adjustment, Formulation change, Initiation/discontinuation and IV-to-oral transition timing.

#### (d) Medication reconciliation

Medication reconciliation was assessed by nurses and pharmacists for patients undergoing total thyroidectomy during the perioperative period. This was done to help ensure patients’ medication history was as accurate as possible and that they were establishing clear continuity of care with respect to calcium and vitamin D supplementation while transitioning through levels of care. This collaborative approach between nursing and pharmacy staff ensured comprehensive medication verification.

#### (e) Collaborative ward rounds

Multidisciplinary collaborative ward rounds were conducted three times weekly, comprising head and neck surgeons, nurses, and clinical pharmacists. During these rounds, healthcare providers systematically reviewed each patient’s calcium and PTH levels, current supplementation regimen, and readiness for transition from intravenous to oral therapy. They also discussed perioperative medication management for patients undergoing total thyroidectomy and provided consultations for individual patients in accordance with the institutional protocol. These multidisciplinary rounds facilitated continuous communication and shared decision-making among the entire care team.

This retrospective observational cohort study received ethical approval (No. TREC2025-KY-136) from the Institutional Review Board of Beijing Tongren Hospital, which granted a waiver of informed consent. The study analyzed existing, de-identified data from routine clinical care and an institutional quality improvement initiative, without involving any prospective experimental interventions. All procedures were performed in accordance with the Declaration of Helsinki.

### Data collection

Data was collected for both groups, and included: demographic and clinical characteristics of each subject (age, gender, and comorbidities); patient hospitalisation related information (date of admission, date of discharge, length of stay, primary and discharge diagnoses); details about the surgical procedure (type of surgical procedure, length of operation); perioperative laboratory tests (serum calcium and parathyroid hormone levels obtained at pre-defined time points); pharmacotherapeutic management of Hypocalcemia and hypoparathyroidism with calcium supplements and active vitamin D analogue, to include type of calcium supplement and active vitamin D analogues used; dosing information regarding calcium supplements dosage, length of therapy, administration method, timing of transition from IV to oral supplementation, and time of patient criteria change for discontinuation.

This study evaluated non-guideline-concordant prescribing of calcium and vitamin D analogues in two groups of patients according to the perioperative hypocalcemia management protocol. Non-guideline-concordant prescribing of calcium and vitamin D analogues was defined as the presence of any of the following: i) Calcium use without indication: administration of calcium to low-risk patients (corrected calcium ≥2.10 mmol/L and PTH ≥1.6 pmol/L) without symptoms; ii) Therapeutic omission: failure to administer calcium to high-risk or moderate-risk patients per protocol; iii) Inappropriate oral formulation: use of calcium carbonate in patients with PPI or H_2_-blocker therapy or patients with a history of gastric bypass surgery; iv) Inappropriate calcium or vitamin D analogue dosage: deviation >25% from protocol-specified dose for >24 h; v) Delayed IV-to-oral switch: continuation of IV calcium >24 h after meeting transition criteria.

In order to find factors that may independently be associated with NGC-CaVD, multivariable binary logistic regression was used. Variables that had a p-value <0.10 in univariate analysis or were clinically significant were included in the model. Results are reported as adjusted odds ratios with corresponding 95% confidence intervals. For all data analyses a two-tailed p-value of less than 0.05 was considered statistically significant.

### Study outcomes

The primary outcome of this study was the incidence of non-guideline-concordant prescribing of calcium and vitamin D analogues (NGC-CaVD). The institution’s adherence to clinical pathways was evaluated by assessing the following specific prescription behaviours: providing calcium without an established indication; omitting calcium when an indication was present; providing inappropriate oral formulations of calcium; providing inappropriate dosages of calcium; delaying the transition from IV to oral calcium; and providing inappropriate dosages of vitamin D analogues. The Secondary outcomes included the perioperative costs of calcium and vitamin D analogues. The safety outcomes of this study were the frequency of symptomatic hypocalcemia and the average length of hospital stay.

### Statistical analysis

The cohort that received the multifaceted intervention was termed the intervention group. The cohort treated prior to the implementation of the management protocol (pre-intervention) and those treated after were analyzed for descriptive statistics on the following baseline variables: demographic characteristics, body mass index (BMI), number of comorbidities, whether central lymph node dissection was performed, and surgical time.

For the statistical analyses, we used SPSS Statistics version 26.0. We first used the Shapiro-Wilk test to determine if individual continuous variables passed a normality test. Continuous data that followed a normal distribution were described as mean ± standard deviation and were analyzed with an independent Student’s t-test. Continuous variables that did not follow a normal distribution were summarized using median/interquartile ranges and were analyzed with a Mann-Whitney U-test. Categorical variables were expressed as frequencies and percentages; differences between groups were evaluated using either the Pearson Chi-square or Fisher’s exact tests.

## Results

### Patient characteristics

**FIGURE 2:**
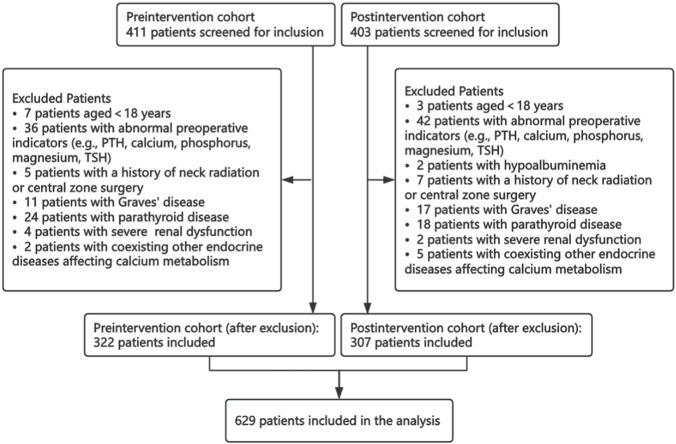
Patient selection flow chart.

A total of 629 patients undergoing total thyroidectomy met inclusion criteria during the study; 322 belonged to the pre-intervention group and 307 were in the post-intervention group as illustrated in the selection flow diagram (Figure 2). There were no statistically significant differences in baseline demographic/clinical characteristics between the two groups; both groups had similar age, sex, BMI, number of comorbidities, central neck dissection rates, and lengths of surgery (Table 1).

**TABLE 1:** General characteristics of study population.

Characteristics	Pre-intervention (n = 322)	Post-intervention (n = 307)	*p*-Value
Age (years), median (IQR)	40.5 (35.25,50.5)	41 (28.25,47)	0.48
Sex (male)	112 (34.78%)	97 (31.60%)	0.40
BMI	23.86 ± 4.16	23.64 ± 4.27	0.52
Total number of comorbidities, median (IQR)	0 (0,1)	0 (0,1)	0.88
Received central neck dissection	286 (88.82%)	281 (91.53%)	0.25
Surgical duration (min)	252.01 ± 87.16	245.63 ± 79.29	0.43

Abbreviations BMI, body mass index; IQR, interquartile range.

### Appropriateness of calcium and vitamin D analogue prescribing

The multifaceted intervention increased the appropriateness of prescribing practices for both calcium and vitamin D analogue compared to pre-intervention level across all evaluated criteria (Table 2). In the pre-intervention group, 13.98% of patients who were not indicated calcium and vitamin D analogue therapy received both medications, compared to 10.75% in post-intervention group (*p* = 0.22). For patients indicated for calcium therapy, only 2.17% in the pre-intervention group did not receive these two medications, compared to 0.65% in the post-intervention group (*p* = 0.11). Inappropriate selection of oral calcium agen decreased from 8.07% before the intervention to 2.93% after the intervention (*p* < 0.01). Inappropriate dosing of both calcium and vitamin D analogues also decreased dramatically, from 23.91% to 11.73% for calcium (*p* < 0.01) and from 30.75% to 6.84% for vitamin D analogues (*p* < 0.01). Under clinical indications, the incidence of failure to transition from intravenous calcium supplementation to oral calcium supplementation in a timely manner decreased from 4.97% before the intervention to 1.95% after the intervention (*p* = 0.04). When evaluated against all six standards for appropriateness (calcium without indication, omission of calcium when indicated, selection of calcium formulation, dosing of calcium, dosing of vitamin D analogue, transition from IV to oral), the proportion of patients experiencing any NGC-CaVD in the post-intervention group was approximately half that of the pre-intervention group (45.96% vs. 23.45%, *p* < 0.01).

**TABLE 2:** Percentage of NGC-CaVD for each and all criteria^a^.

Variable	Pre-intervention (n = 322)	Post-intervention (n = 307)	*p*-Value
Calcium and vitamin D analogue use without indication^b^	45 (13.98%)	33 (10.75%)	0.22
Therapeutic omission of calcium and vitamin D analogue despite indication^b^	7 (2.17%)	2 (0.65%)	0.11
Selection of oral calcium agent	26 (8.07%)	9 (2.93%)	<0.01
Calcium dosage	77 (23.91%)	36 (11.73%)	<0.01
Vitamin D analogue dosage	99 (30.75%)	21 (6.84%)	<0.01
IV to oral	16 (4.97%)	6 (1.95%)	0.04
All 6 criteria	148 (45.96%)	72 (23.45%)	<0.01

Abbreviations: IV, intravenous injection; NGC-CaVD, non-guideline-concordant prescribing of calcium and vitamin D analogues.

^a^ Given that the inpatient pharmacy stocked only oral calcitriol capsules, with no alternative formulations (such as injectable calcitriol) available, the appropriateness of vitamin D analogue formulation selection was not a relevant aspect of prescribing that could be assessed in this study.

^b^ A predefined order set containing calcium and vitamin D analogues was embedded within the hospital information system used by our department of head and neck surgery. Consequently, throughout the study period, these two medications were invariably prescribed together, or omitted together, in clinical practice.

### Root-cause analysis of inappropriate prescribing

The root-cause analysis of inappropriate prescribing revealed distinct patterns of contributing factors between the two cohorts. In the pre-intervention cohort, physician-related factors accounted for the largest proportion (46.30%). The primary issues included: absence of standardized protocols led some surgeons to routinely prescribe prophylactic calcium and vitamin D analogues for all patients within the first 3 postoperative days based on clinical experience, rather than stratified risk assessment; and failure to adjust medication dosages according to dynamic changes in serum calcium and parathyroid hormone (PTH) levels. Pharmacist-related factors constituted the second largest category (31.11%), primarily attributable to the absence of a protocol-based intervention framework and inadequate pharmacy staffing during weekends and holidays, which resulted in gaps in pharmaceutical care. Nursing-related factors accounted for 12.96%, mainly stemming from suboptimal communication channels that delayed the timely reporting of symptomatic hypocalcemia to the medical team. Patient-related factors comprised 9.63%, predominantly involving impaired absorption of oral calcium carbonate due to prior gastric bypass surgery or concurrent use of proton pump inhibitors or H_2_-receptor antagonists.

In the post-intervention group, physician-related factors remained the primary cause (51.40%), with the key issue shifting to over-prophylactic prescribing driven by excessive concern for postoperative hypocalcemia risk despite no clear indications. Pharmacist-related factors increased to 40.19%, attributable to the interruption of pharmaceutical intervention due to the lack of weekend/holiday pharmacist coverage. Patient-related factors decreased to 8.41%, which primarily involved the temporary initiation of PPIs or H_2_-receptor antagonists during the postoperative period. Nurse-related factors were reduced to 0%.

### Calcium and vitamin D analogues cost

The multifaceted intervention resulted in a significant reduction in medication expenditures. Per capita medication costs for calcium and vitamin D analogues decreased from RMB 45.72 (22.72, 53.28) in the pre-intervention group to RMB 34.38 (23.71, 32.69) (*p* < 0.01), while *per capita* costs associated with NGC-CaVD decreased from RMB 17.34 (0, 19.56) to RMB 4.42 (0, 0) (*p* < 0.01) (Table 3).

**TABLE 3:** Per capita costs of calcium and vitamin D analogues.

Variable	Pre-intervention (n = 322)	Post-intervention (n = 307)	*p*-Value
Per capita cost of CaVD (yuan), median (IQR)	45.72 (22.72, 53.28)	34.38 (23.71, 32.69)	<0.01
Per capita cost of NGC-CaVD (yuan), median (IQR)	17.34 (0, 19.56)	4.42 (0, 0)	<0.01

Abbreviations CaVD, calcium and vitamin D analogues; IQR, interquartile range; NGC-CaVD, non-guideline-concordant prescribing of calcium and vitamin D analogues.

### Incidence of symptomatic hypocalcemia and length of hospital stay

There was no statistically significant difference in the incidence of symptomatic hypocalcemia (6.83% vs. 5.86%, *p* = 0.62) or in the length of hospital stay (7.16 ± 2.70 vs. 7.21 ± 2.65, *p* = 0.75) between the pre- and post-intervention cohorts (Table 4).

**TABLE 4:** Clinical safety outcomes.

Variable	Pre-intervention (n = 322)	Post-intervention (n = 307)	*p*-Value
Symptomatic hypocalcemia, n (%)	22 (6.83%)	18 (5.86%)	0.62
Average length of hospital stay (days)	7.16 ± 2.70	7.21 ± 2.65	0.75

### Factors influencing NGC-CaVD

A multivariable binary logistic regression analysis was performed to identify independent factors associated with the occurrence of NGC-CaVD in the intervention group. The analysis found that a longer operative time and longer length of stay were significantly associated with a higher risk of NGC-CaVD (*p* < 0.05 for each). However, there were no significant associations observed between NGC-CaVD and patient sex, age, BMI, number of comorbidities or performance of central lymph node dissection (Table 5).

**TABLE 5:** Multivariable analysis of factors associated with NGC-CaVD in the post-intervention group.

Variable	Adjusted OR (95% CI)	*p*-Value
Gender (male)	1.099 (0.595–2.032)	0.763
Age (years)	0.998 (0.976–1.021)	0.879
BMI	0.941 (0.877–1.010)	0.941
Total number of comorbidities	1.089 (0.824–1.440)	0.549
Received central neck dissection	1.223 (0.431–3.468)	0.706
Surgical duration	1.012 (1.007–1.035)	0.023
Length of hospital stay	1.128 (1.008–1.262)	0.036

Abbreviations: BMI, body mass index; NGC-CaVD, non-guideline-concordant prescribing of calcium and vitamin D analogues.

## Discussion

This study demonstrates that implementing a multifaceted intervention in a head and neck surgical population increased the appropriateness of perioperative hypocalcemia management following total thyroidectomy and decreased the prescribing cost of calcium and vitamin D analogues. The primary result was a statistically significant decrease overall in NGC-CaVD between pre-intervention and post-intervention time periods. This finding was primarily driven by significant reductions in specific prescribing errors, specifically inappropriate dosages of calcium and vitamin D analogue, inappropriate selections of oral calcium preparations, and failure to promptly transition from intravenous to oral supplementation when clinically indicated. Although a decreasing trend was noted in other NGC-CaVD categories, such as calcium use without indication and therapeutic omission when an indication existed, these did not reach significance. There was, however, no significant difference in the incidence of symptomatic hypocalcemia in either group following the intervention. This suggests that whilst attempts were made to standardize pharmacotherapy with a pre-developed protocol, the intervention was not at the expense of patient safety. In addition, the average length of hospital stay indicated that the intervention did not result in an unintended length of stay.

The unique aspect of this study centered on developing and evaluating a coordinated intervention package containing five main components: guideline establishment, continued education, proactive medication review, medication verification, and multidisciplinary ward rounds. All five components jointly afforded three paradigms in the management of hypocalcemia: moving the management of hypocalcemia from off label or empiric to standardized evidence-based care; changing from unilateral decision making to multidisciplinary collaborative processes; and finally moving from static, one-time interventions to dynamic, closed loop quality assurance.

Drawing upon our multi-focal intervention, there was a significant reduction in the proportion of NGC-CaVD from 45.96% before the intervention to 23.45% post-intervention. This improvement corresponds to, if not exceeds, the outcomes provided in previous bundled studies. For example, Lechner et al. found that a multi-component bundle aimed at changing management practices could increase adherence to the protocol to monitor postoperative calcium levels in 10.9% of patients, and achieve a 78.3% adherence to guideline-recommended treatment for hypocalcemia (Lechner et al., 2023). Similarly, Gonsalves et al. reported a reduction of 20% of patients receiving additional doses of intravenous calcium supplementation after implementing a similar standardized clinical pathway for managing hypocalcemia following a total thyroidectomy (Gonsalves et al., 2020). The significant therapeutic effect observed in the present study may be attributed to the synergistic interaction of multiple core components in an intervention package, which as a whole created a progressive management system of “institution, personnel, process, and quality control.” Clinical guidelines provided standardized, evidence-based support across the continuum of the pathway to care; Education promoted a common awareness about, and engagement of the guidelines, between surgical teams to resolve knowledge-practice gaps; Pharmacists contributed to the implementation of the guidelines as evidenced by medication verification, proactive reviewing patients and making recommendations integrated into care rounds, and dynamic processes of a collaborative ward round. This design ensured macroscopic standardization without compromising microscopic flexibility, ultimately creating a complementary partnership between pharmaceutical expertise and surgical practice.

Adoption of the management protocol after intervention led to a significant increase in satisfying the timely transition from intravenous to oral calcium. This result is consistent with existing evidence. The study conducted by Babonji et al., emphasizes that timely conversion from intravenous to oral reduces risk of catheter-related infections and workload for healthcare staff (Babonji et al., 2021). Clinical barriers exist to introducing or routinely managing that transition; and it is common practice internationally, but have not been widely adopted in the clinical practice of hypocalcemia management in China, likely due in part, to the general awareness of clinicians to the feasibility or benefits of the transition in the existing context. The data from the pre-intervention group of this study indicate that a proportion of patients experienced a delay in switching from intravenous to oral formulations, highlighting shortcomings in clinical practice. In addition, the surgical team noted that a significant barrier to transitioning patients orally to oral treatment was related to the general belief of the team regarding oral calcium formulations being far less bioavailable than intravenous preparation and they doubted the treatment effectiveness as a result. The second general barrier highlighted was the significant overall burden of clinical workload in a head and neck surgery department; either in groups or individually, clinicians may often find themselves precluded from in time identifying patients who could transition timely to oral therapies.

To address the barriers to switching from intravenous to oral administration, our institution developed a guideline that emphasized a risk stratified approach based on serum calcium level and clinical symptoms. Specifically, patients in the cohort where serum calcium was below 1.9 mmol/L or who exhibited severe symptoms of hypocalcemia were given intravenous calcium replacement to expedite a more rapid correcting. For patients who maintained serum calcium at or above 1.9 mmol/L without severe symptoms of hypocalcemia, oral calcium supplementation was recommended in order to standardize protocol and promote timely transition. This approach effectively balances the need for timely correction in acute cases with the benefits of early oral treatment in clinically stable patients. Additionally, our intervention created a collaborative model where clinical pharmacists worked along-side surgical colleagues to proactively identify candidates and recommend switch during a structured collaborative clinical rounds. This multidisciplinary strategy proved effective, successfully reducing the rate of untimely IV-to-PO to 1.95% in the post-intervention cohort.

Our root-cause analysis yielded granular insight into the inappropriate prescribing before and after the intervention. While the multifaceted intervention successfully addressed systemic clinical practice gaps, such as the absence of standardized guidelines and knowledge-practice discrepancies, and eliminated nurse-related communication errors, it also highlighted the persistent challenges posed by human factors. Notably, physician-related factors shifted from a lack of evidence-based knowledge and guideline familiarity to an overly cautious clinical mindset in prescribing decisions. This finding suggests that future management strategies could incorporate additional clinical decision-making factors, such as surgical duration, to implement more refined stratified patient management, thereby alleviating physicians’ unwarranted clinical concerns (Jan et al., 2024; Chen et al., 2021). Similarly, the persistent impact of gaps in pharmacist coverage identifies a structural limitation that requires institutional-level solutions, such as extending pharmacy services to cover holidays and weekends, to achieve full protocol adherence in the perioperative management of post-thyroidectomy hypocalcemia.

This study demonstrates that implementing a multifaceted intervention significantly reduced the medication costs for calcium and vitamin D analogues, with expenditures specifically attributable to non-guideline-concordant prescribing decreasing substantially from RMB 17.34 to RMB 4.42 per patient. Furthermore, the *per capita* medication costs for calcium and vitamin D analogues decreased from RMB 45.72 to RMB 34.38, representing a 24.80% reduction in drug expenditure. Based on the annual admission volume of 629 patients undergoing total thyroidectomy in our institution, this intervention is estimated to save approximately CNY 7132.86 in annual medication costs for calcium and vitamin D analogues. This downward trend in medication costs is consistent with the conclusions of previous similar quality improvement studies on perioperative medication management after thyroidectomy (Singer et al., 2012; Nicholson et al., 2020). The significant reduction in medication costs is primarily attributable to the marked decrease in the incidence of NGC-CaVD after the intervention, which further led to rational clinical adjustments including timely optimization of calcium and vitamin D analogue dosages, shortening of unnecessary medication courses, and an increased proportion of transition from intravenous to oral formulations. These optimized medication strategies effectively reduced the waste of pharmaceutical resources caused by non-guideline-concordant prescribing, achieving the goal of value-based care that balances clinical rationality and economic efficiency in perioperative management.

In our analysis of the present intervention, no significant clinical difference was observed between the two groups in terms of symptomatic hypocalcemia or the duration of hospital admission. This suggests that the multifaceted intervention implemented in this study successfully achieved the standardization of perioperative care for patients undergoing total thyroidectomy without impact symptomatic hypocalcemia and length of stay, a finding consistent with the results reported by Roy et al. (2025). It also aligns with previous trials involving multifaceted interventions, which indicate that structured care pathways can improve prescribing behavior without adversely affecting key clinical outcomes (Patel et al., 2018). Furthermore, the reason for finding no significant clinical difference in the occurrence of symptomatic hypocalcemia in this study may be attributed, in part, to the low baseline rates of this adverse event in this population and the limitations imposed by the small number of patients recruited for this study.

While most studies support using postoperative PTH levels as a basis for patient risk stratification, many factors can affect the interpretation of PTH results, including the timing of blood sample collection after surgery (Samargandy et al., 2023; Mattoo et al., 2021). In clinical settings where strict adherence to sample timing is often challenging, implementing strict adherence to PTH as a predictive marker may not be feasible. Therefore, considering operational realities specific to our institution, we adopted a dual marker approach to patient risk stratification, utilising serum calcium and PTH values, for more manageable implementation while still maintaining a patient-centred management strategy.

In order to assist in refining our intervention strategy in the future, we performed a multivariate analysis to examine potential factors related to NGC-CaVD. We believe that this study may be one of the first studies to identify these variables in the context of a structured quality improvement initiative. The analysis demonstrated that longer operative time and increased post-operative hospitalisation were independent variables associated with a higher likelihood of developing NGC-CaVD. Previous studies have confirmed the correlation between longer operative time in thyroidectomy and increased prescription rates of calcium and vitamin D analogs (Albuja-Cruz et al., 2015; Jullamusi et al., 2025). Based on our informal discussions with head and neck surgeons during the implementation of this management, we speculate that the association between surgery duration and supplement prescription rates may stem from surgeons’ clinical considerations: to avoid the risk of hypocalcemia associated with prolonged surgery, they may adopt a more proactive supplementation strategy, which leads to increased prescription rates of calcium and vitamin D analogues, even if these prescriptions do not fully align with guideline recommendations. Previous studies have confirmed the correlation between surgery duration and postoperative hypocalcemia (Cao and Wu, 2025; van Kinschot et al., 2023), suggesting that in cases of complex or prolonged surgery, clinical decision-making may involve special circumstances not fully addressed by the guidelines. Therefore, although our current multi-faceted interventions have generally standardized care procedures, they may lack sufficient flexibility to handle such complex cases. Based on these findings, we believe that intervention strategies need to be more adaptive. We are currently adjusting our approach and plan to incorporate decision support tools tailored for different levels of surgical complexity to further optimize clinicians’ prescribing practices and patient outcomes.

There are numerous limitations that should be taken into account when interpreting this study’s findings. Firstly, the retrospective, observational nature of this study may limit the generalisability of the findings to other hospital settings; additionally, most of the data were retrieved from electronic medical records, and inaccuracies regarding the appropriateness of Hypocalcemia therapy may exist due to incomplete or unclear documentation. In addition, we did not take into account potential confounding variables, such as types of thyroid cancer differentiation, surgeon experience and rank, which may influence perioperative practices for hypo-calcaemia. Finally, the relatively short duration of follow-up and sample size may limit the generalisability of the findings to the overall population. Prospective, multi-centre studies are needed for further validation and to extend the findings of our research.

## Conclusion

The findings of this study demonstrate that the implementation of a multifaceted intervention, involving the establishment of a standardised management protocol, comprehensive education for all healthcare workers, proactive review of medications by pharmacists and collaborative approaches through physician-pharmacist ward rounds, significantly increased the appropriateness of prescribing calcium and vitamin D analogue therapy in patients undergoing total thyroidectomy. The multifaceted intervention reduced both the frequency and cost of NGC-CaVD prescribing, leading to more consistent and standardized postoperative patient care. Although the integrated approach was successful in our institution, it will be essential to continue further explorations toward determining the sustainability of this intervention and developing strategies for further refinement of the pathway. Future studies should focus on continuing to adapt and validate the pathway within diverse patient populations and healthcare environments to develop its utility and practical implications beyond this study’s participants.

## Data Availability

The raw data supporting the conclusions of this article will be made available by the authors, without undue reservation.
